# Unraveling psilocybin’s therapeutic potential: behavioral and neuroplasticity insights in Wistar-Kyoto and Wistar male rat models of treatment-resistant depression

**DOI:** 10.1007/s00213-024-06644-3

**Published:** 2024-07-04

**Authors:** Magdalena Kolasa, Agnieszka Nikiforuk, Agata Korlatowicz, Joanna Solich, Agnieszka Potasiewicz, Marta Dziedzicka-Wasylewska, Ryszard Bugno, Adam Hogendorf, Andrzej Bojarski, Agata Faron-Górecka

**Affiliations:** 1 Department of Pharmacology, Maj Institute of Pharmacology Polish Academy of Sciences, Kraków, Poland; 2 Department of Behavioral Neuroscience & Drug Development, Maj Institute of Pharmacology Polish Academy of Sciences, Kraków, Poland; 3 Department of Medicinal Chemistry, Maj Institute of Pharmacology Polish Academy of Sciences, Kraków, Poland

**Keywords:** Treatment-resistant depression, Wistar Kyoto rats, Psilocybin, Neuroplasticity, Brain-derived neurotrophic factor (BDNF), Activity-regulated cytoskeleton-associated protein (Arc), Social interaction, Weight gain, Long-term effect

## Abstract

**Rationale:**

Our study aimed to unravel the unknown mechanisms behind the exceptional efficacy of Psilocybin (PSI) in treating treatment-resistant depression (TRD). Focusing on Wistar-Kyoto (WKY) rats with a TRD phenotype and Wistar (WIS) rats as a normative comparison, we investigated behavioral and neuroplasticity-related responses to PSI, striving to shed light on the distinctive features of its antidepressant effects.

**Objectives:**

We set out to assess the behavioral impact of acute and prolonged PSI administration on WKY and WIS rats, employing Novel Object Recognition (NORT), Social Interaction (SI), and Forced Swimming Test (FST). Our secondary objectives involved exploring strain-specific alterations in neuroplasticity-related parameters, including brain-derived neurotrophic factor (BDNF) and activity-regulated cytoskeleton-associated protein (Arc).

**Methods:**

Conducting post-acute and extended assessments after a single PSI administration, we applied behavioral tests and biochemical analyses to measure serum BDNF levels and neuroplasticity-related parameters in the prefrontal cortex. Statistical analyses were deployed to discern significant differences between the rat strains and assess the impact of PSI on behavioral and biochemical outcomes.

**Results:**

Our findings uncovered significant behavioral disparities between WKY and WIS rats, indicating passive behavior and social withdrawal in the former. PSI demonstrated pronounced pro-social and antidepressant effects in both strains, each with its distinctive temporal trajectory. Notably, we identified strain-specific variations in BDNF-related signaling and observed the modulation of Arc expression in WKY rats.

**Conclusions:**

Our study delineated mood-related behavioral nuances between WKY and WIS rat strains, underscoring the antidepressant and pro-social properties of PSI in both groups. The distinct temporal patterns of observed changes and the identified strain-specific neuroplasticity alterations provide valuable insights into the TRD phenotype and the mechanisms underpinning the efficacy of PSI.

**Supplementary Information:**

The online version contains supplementary material available at 10.1007/s00213-024-06644-3.

## Introduction

Psychedelic compounds, notably psilocybin (PSI), have garnered considerable attention in recent clinical research, especially in the field of major depressive disorder (MDD) and particularly in treatment-resistant depression (TRD). In numerous clinical trials, the administration of psilocybin (PSI) has demonstrated enduring reductions in symptoms of depression and anxiety, often with notable therapeutic effects observable after just one or two doses (Dos Santos et al. 2016; Carhart-Harris et al. 2018; Gregorio et al. 2021; Goodwin et al. 2023). As a psychoactive compound, PSI profoundly influences various mental domains, including sensory perception and thought processes (Halberstadt et al., 2011). These properties, coupled with its potential to enhance cognitive functions, position psilocybin as a promising candidate for MDD treatment, given the close association between cognitive symptoms and depression-related impairment (Doss et al. 2021). Interestingly, emerging findings propose the potential dissociation of the hallucinogenic properties of psychedelic substances from their antidepressant-like and neuroplasticity-enhancing effects (Cameron et al. 2021; Hesselgrave et al. 2021), suggesting the feasibility of identifying compounds or therapeutic combinations capable of preserving the antidepressant attributes of psychedelics while circumventing the hallucinogenic side effects. Nonetheless, the precise mechanisms underlying these distinct effects remain unclear.

**Fig. 1:**
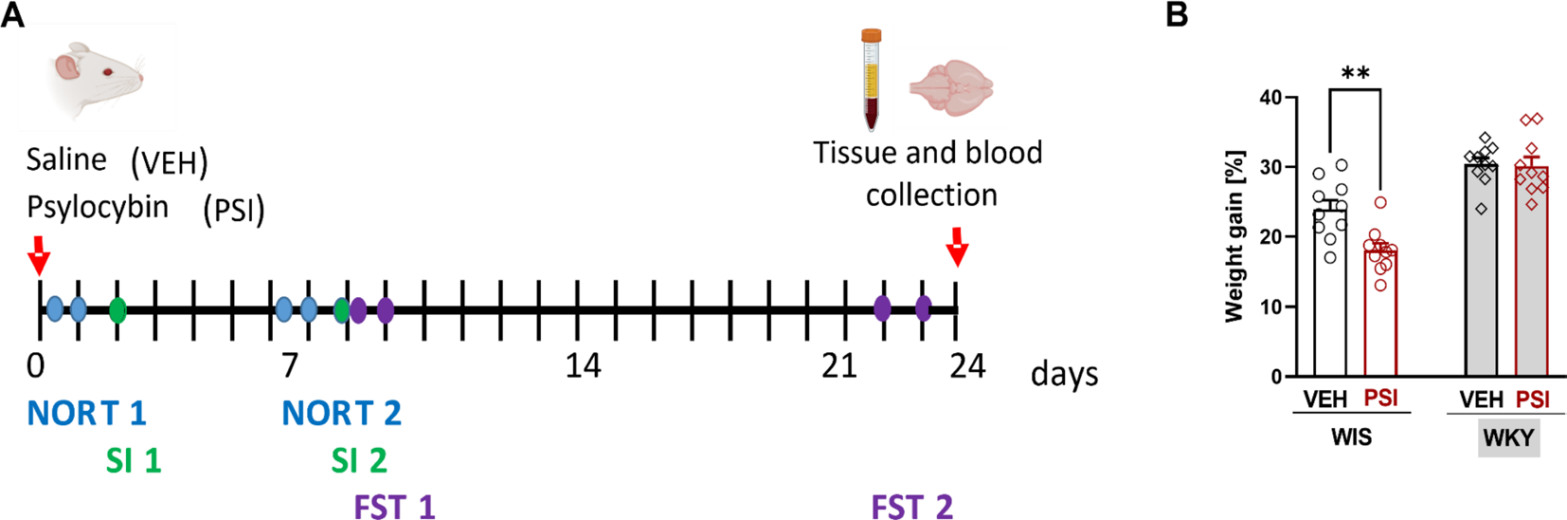
(**A**) Experimental paradigm presenting the timeline of behavioral procedures and biochemical analyses. The behavioral procedures spanned over 23 days, and on the 24th day, animals were sacrificed, and tissue was collected for biochemical analyses. (**B**) Effect of psilocybin on weight gain. Body weight was measured on the day of PSI administration and 23 days after PSI administration. WKY rats are depicted as rhomboid symbols and grey shaded bars, while WIS rats are represented as squared symbols and white bars. In WIS rats, there was a significant decrease in body weight gain [%] compared to the vehicle-treated group (**p < 0.01, two-way ANOVA followed by Tukey’s multiple comparison test)

As the primary considerations regarding the rapid and sustained antidepressant action of psilocybin predominantly pertain TRD, we employed our studies in Wistar Kyoto (WKY) rats, a well-validated model of TRD (Willner et al. 2019; Papp et al. 2018). WKY rats endogenously exhibit behavioral abnormalities that parallel some manifestations of major depressive disorder (MDD), including passive coping strategies, heightened physiological and behavioral responses to stress, encompassing anxiety and depressive-like behaviors (such as an exaggerated stress response, increased learned helplessness, anhedonia, neophobia, and reduced social interactions), neuroendocrine and HPA axis dysregulation, alterations in monoaminergic and glutamatergic systems, neuroinflammation, and perturbed neurogenesis (as reviewed in Millard et al. 2020). It has been showed that WKY rats spent significantly more time immobile than Wistar rats in the forced swim test (FST) (Lahmame and Armario 1996). Although this test is widely used to study the antidepressant activity of drugs, it has faced increasing criticism, which emphasizes that the measured immobility does not necessarily indicate despair or depression, but may rather be a strategic, adaptive behavior aimed at conserving energy in a stressful situation (Castagné et al. 2011; Molendijk and de Kloet 2015). It is therefore important to consider that the FST may in fact assess coping strategies rather than depressive states. Furthermore, although the FST has been instrumental in characterizing a strain of WKY rats as exhibiting depression-like behaviour due to their increased immobility (Paré 1994), recent critiques have questioned the direct correlation of FST scores with depressive behaviour, arguing that the observed immobility may reflect broader psychobiological strategies rather than specific depressive symptoms (Commons et al. 2017; Campus et al. 2015). This strain has also been shown to have reduced performance on the Novel Object Recognition Test (NORT) (Shoval et al. 2016) and reduced social interactions (Nam et al. 2014); detailed described in Aleksandrova et al. 2019. Moreover, investigations utilizing acute doses of conventional antidepressants in the FST, or chronic drug administration in the chronic mild stress (CMS) model, have indicated that WKY rats do not exhibit a response to conventional antidepressants, except for ketamine or deep brain stimulation (Willner et al. 2019). Hence, WKY rats, as an endogenous model of TRD, merit special attention in the quest for novel targets in preclinical research (Kolasa and Faron-Górecka 2023) and, more broadly, in advancing our comprehension of TRD. As WKY rats exhibited altered behavior in response to a single PSI dose, demonstrating distinct outcomes in depression- and anxiety-related parameters (Hibicke et al. 2020, 2023), we conducted behavioral measurements focusing on certain aspects of rats’ behavior. The assessments included the NORT, FST, Social Interaction (SI) test, performed at various time points (Fig. 1A) following the single exposure to PSI. TRD patients often exhibit deficits in attention, memory, executive function, and processing speed (Vancappel et al. 2023). The behavioral tests we selected for the present study reflect major aspects of the behavioral deficits observed clinically and depend on the function of specific neurocircuits disturbed in TRD patients. There is strong evidence suggesting that the prefrontal cortex, hippocampus, and amygdala are key elements in neural circuits significantly impacting cognitive impairment in depression (Drevets et al. 2008; Li et al. 2015; Chan et al. 2016; Yang et al. 2021a, b). It has been shown that both cortical and hippocampal areas are often reduced in depressed patients (Drevets et al. 2008). Furthermore, post-mortem studies of patients and animal models of depression have shown that neuronal atrophy in these brain regions plays a key role in the pathophysiology of cognitive dysfunction in MDD. Disturbances in the neural circuitry associated with TRD may be linked to cognitive tasks dependent on hippocampal function, such as object localization and pattern discrimination (Yang et al. 2021b). Additionally, areas of the frontal lobes that are crucial for functions such as novel object recognition and spontaneous alternation also show changes after PSI treatment. It has been identified that PSI improves cognitive flexibility in response to activity anorexia, which may be related to 5-HT1A and 5-HT2A receptor mechanisms (Conn et al. 2024). NORT and SI tests likely engage brain circuits relevant to TRD, including the hippocampus (key for memory and spatial processing), as well as the amygdala, prefrontal cortex, and mesolimbic dopamine system involved in cognitive functioning and social behaviors. Encouragingly, we aimed to check if the changes in behavior after PSI treatment will be paralleled with biochemical changes in the brain. In our study, we decided to focus on the prefrontal cortex, as this brain region appears to be significantly affected by psilocybin treatment in humans (Carhart-Harris et al. 2012). Recent findings also reveal that PSI significantly affects network organization in the rat cortex (Silverstein et al. 2024). On molecular level, it has been shown that PSI induces a sustained increase in dendritic spines in the frontal cortex, which may impact the brain’s adaptive capacity (Shao et al. 2021). It is believed that psilocybin may contribute to new therapeutic strategies for treating TRD, based not only on its antidepressant properties but also on its stimulation of neuroplasticity (Magaraggia et al. 2021; Calder and Hasler 2023). Thus, after behavioral procedures, we performed biochemical analyses focusing on neuroplasticity-related targets. In our approach, we focused on the expression of BDNF and its receptors (NTRK2, NTRK1, and NGFR), alongside other neurotrophins (NGF, NT3, and NT4), which are part of the broader neurotrophic signaling network. Based on extensive literature, a decrease in BDNF levels has been observed in the serum and brain samples of patients (Karege et al. 2002; de Azevedo Cardoso et al. 2014; Cavaleri et al. 2023), as well as in individuals who died by suicide (Khan et al. 2019). Conversely, antidepressant treatment increases BDNF expression in the brains of depressed patients (Dwivedi 2009; Cavaleri et al. 2023). Studies on baseline BDNF levels in the WKY strain are inconclusive. Some studies have shown lower baseline BDNF levels in the serum of WKY rats compared to WIS rats (Kyeremanteng et al. 2012), while others have reported no changes (Kaadt et al. 2022). Among neurotrophin receptors, Trkβ is the primary receptor for BDNF and is integral to the neuroplastic changes associated with antidepressant effects (Minichiello 2009). While the TrkA is traditionally associated with Nerve Growth Factor (NGF), is believed to interact with BDNF and NT4 pathways as well, suggesting a broader role in depression’s neurobiology (Huang and Reichardt 2001; Tessarollo and Yanpallewar 2022). Interestingly, recent studies have indicated that the antidepressant effects of psilocybin may be attributed to their direct binding to the tyrosine receptor kinase β (Trkβ), which exhibits an affinity approximately 1,000 times greater than that of conventional antidepressants (Moliner et al. 2023). Additionally, it has been demonstrated that the impact of psychedelics on neurotrophic signaling, plasticity, and antidepressant-like behavior in mice is contingent upon Trkβ binding and the facilitation of endogenous BDNF signaling while remaining independent 5-HT2A activation (Ly et al. 2018). In our study, besides neurotrophins and its receptors, we assessed additional molecular targets known as markers of neuroplasticity, such as Arc, Gria1, Gria2, PSD95, St8sia2, and St8sia4. While not directly part of the canonical BDNF signaling pathway, these targets play roles in regulating synaptic structure, function, and plasticity-related signaling pathways. They may be influenced by BDNF signaling and could serve as potential targets for the action of PSI in WKY and WIS rats. Moreover, these targets may indicate adaptive changes in neural networks correlated with improvements in depressive and anxiety-related symptoms.

## Methods

### Experimental design

The timeline of behavioral tests, including the Novel Object Recognition Task (NORT), Social Interaction (SI) test, and Forced Swim Test (FST), is presented in the schematic schedule of the experiments (Fig. 1A). The acquisition phase of the NORT test was conducted 4 h and 6 days after drug administration. The SI test was conducted 2 and 8 days after drug administration. The FST was performed 9 and 23 days after drug administration. The NORT and SI tests were scheduled before the first FST to minimize the potential confounding effects of stress from the FST on cognitive and social behaviors. By administering the FST later, we aimed to independently evaluate the effects of psilocybin on mood-related behaviors without the immediate aftermath of cognitive or social tests. However, we acknowledge that the placement of these tests could interact in complex ways, influencing subsequent behavior. For instance, earlier behavioral tests might either sensitize or habituate the animals to testing conditions, which could potentially affect their responses in later tests. Encouragingly, we aimed to check if the changes in behavior after PSI treatment will be paralleled with biochemical changes in the brain.

### Animals

Male Wistar Han rats (7 weeks old, weighing approximately 220 g) and Wistar-Kyoto rats (7 weeks old, weighing approximately 200 g) were used for the experiments. The animals were provided with ad libitum access to food and water and were housed in a controlled environment with a constant room temperature (22 ± 1 °C), humidity-controlled colony rooms, and a 12-hour light/dark cycle (lights on at 7 a.m.). Behavioral testing was conducted during the light phase of the light/dark cycle. The experimental procedures adhered to the European Guidelines for animal welfare (2010/63/E.U.) and were approved by the II Local Ethics Committee for Animal Experiments at the Maj Institute of Pharmacology, Polish Academy of Science, Krakow, Poland (permission number: 256/2022). As there are indications in the literature that PSI can affect body weight (Huang et al. 2022), animals were weighed before and after the behavioral experiments.

### Drugs

Psilocybin was synthesized by AH and RB at the Department of Medicinal Chemistry of the Maj Institute of Pharmacology using the method described by Shirota et al. (2003). The tetrabenzyl pyrophosphate used was prepared according to the procedure described in Organic Syntheses by Nelson et al. (2003). Psilocybin was dissolved in physiological saline (vehicle) and administered intraperitoneally (i.p.) at a single dose of 0.3 mg/kg of body weight. Control animals received an injection of the vehicle. Each experimental group consisted of 10 subjects. Given the goal of choosing the lowest effective dose of PSI with antidepressant potential, a dose of 0.3 mg/kg was determined based on a review of the literature including studies in humans (Ross et al. 2016; Goodwin et al. 2022, 2023) and in animal models (Catlow et al. 2013; Horsley et al. 2018; Jones et al. 2020; Hibicke et al. 2020; Risca 2021; Hesselgrave et al. 2021; Higgins et al. 2021; Hibicke and Nichols 2022). In human clinical trials, PSI doses typically range from 20 to 30 mg per 70 kg of body weight (0.29 to 0.43 mg/kg) (Garcia-Romeu et al. 2021). In animal models, most studies utilize doses equal to or less than 1.0 mg/kg of PSI. Consistent with classical pharmacology approach, the behavioral effects of PSI treatment are dose-dependent, and primarily mediated by the occupation of serotonin receptors, namely 5-HT2A, 5-HT2C, 5-HT1A, and 5-HT7. In a study by Higgins et al. (2021) examining the dose-response effects of PSI on rat behavior, a dose of 0.3 mg/kg was identified as a threshold dose, eliciting some behaviors associated with 5-HT2A receptor activation, but not 5-HT2C or 5-HT1A activation. Additionally, a recent study by Kiilerich et al. (2023) investigated the dose-response relationship of PSI on rat behavior, as well as dose-dependent occupancy of serotonin receptors. The study demonstrated that low doses of PSI did not induce significant changes in wet-back shakes, whereas doses 1 mg/kg and above evoked a significant increase compared to the control group. Furthermore, doses exceeding 1 mg/kg revealed sedative effects of psilocybin. Based on this data, we chose to utilize a dose of 0.3 mg/kg in our study, as it appeared to exhibit antidepressant properties and pro-cognitive effects without inducing overt psychedelic effects.

### Novel object recognition task (NORT)

The NORT was conducted as previously described (Nikiforuk et al. 2013), with tests conducted 4 h and 6 days after PSI administration. Rats were tested in a dimly lit (25 lx) open field made of dull grey plastic (dimensions: 66 × 56 × 30 cm). Prior to testing, each rat was habituated to the arena (without any objects) for 5 min, 24 h before the actual test. The NORT consisted of two 3-minute trials separated by a 24-hour inter-trial interval (ITI). Psilocybin (PSI) or vehicle (VEH) was administered 4 h before the first trial (T1). During the first trial (familiarization, T1), two identical objects (A1 and A2) were placed in opposite corners, approximately 10 cm from the walls of the open field. In the second trial (retention, T2), one of the objects was replaced by a novel one (A = familiar, and B = novel). The objects used included a glass bulb filled with gravel and a plastic bottle filled with sand (NORT 1). In the second test (NORT 2), the set of objects was replaced with a new one (metal can and glass cylinder). The photographs of paired objects were included in the Supplementary Figure S1, panel D. The height of the objects was comparable (~ 12 cm), and they were of sufficient weight to prevent displacement by the animals. Object exploration was defined as looking, licking, sniffing, or touching the object, while leaning against, standing on, or sitting on the object were not considered exploratory behaviors. Rats spending less than 5 s exploring both objects within 3 min at either T1 or T2 were eliminated from the study. Behavior was recorded by a camera placed above the arena and connected to the Any-Maze^®^ tracking system (Stoelting Co., Illinois, USA). An experimenter blinded to the treatment conditions manually assessed the exploration time. Additionally, the distance traveled was automatically measured using the Any-Maze^®^ tracking system. The Discrimination Index (DI) was calculated as DI(EB–EA)/(EA + EB) based on the exploration time (E) of the two objects. The entire NORT procedure was repeated 6 days after the first test, so each rat was tested twice (NORT 1 and NORT 2).

### Social interaction (SI) test

The SI test was performed as previously described (Potasiewicz et al. 2020), with tests conducted 2 days and 8 days after PSI administration. Social behavior was observed in same-treatment pairs of rats placed in a dimly illuminated (15 lx) open field (dimensions: 57 × 67 × 30 cm, material: black Plexiglas). Rats were individually placed in the open field for 5 min one day before the test to adapt them to the testing area. On the test day, two unfamiliar rats of matched body weight (± 5 g) were placed in the open-field arena, and their behaviors were recorded for 10 min using a Sony light-amplification CCD camera placed above the arena and connected to a PC running Noldus MPEG recorder 2.1. An experimenter blind to the treatment conditions analyzed the videos offline using Noldus EthoVision XT, version 14.

Measured social behaviors included sniffing (rat sniffs the body of the conspecific), anogenital sniffing (rat sniffs the anogenital region of the conspecific), social grooming (rat licks and chews the fur of the conspecific), climbing (rat climbs over the back of the conspecific/stands on the back of the conspecific), and following (rat moves toward and follows the conspecific). The duration and number of episodes of social behavior were measured for each rat separately.

### Forced swim test (FST)

A modified FST procedure was used (Detke et al. 1995; Kuśmider et al. 2007). During the pre-test (performed 8 days after PSI administration), rats were forced to swim for 15 min in opaque cylinders (21 cm in diameter), filled with water (23–25 °C) to a depth of 30 cm. They were then removed from the water, dried with towels, placed in a warm enclosure, and returned to their home cages. The cylinders were emptied and cleaned between each rat. Twenty-four hours later (i.e., 9 days after PSI administration), animals were placed in the cylinders filled in the same way as during the pre-test, and their behavior was video-taped. Quantification was done by behavioral sampling, where the 5-minute test session was divided into 60 intervals lasting 5 s each. An experienced observer categorized the behavior as climbing, swimming, or immobility based on the predominant activity in each interval. Climbing behavior was defined as upward-directed movements of the forepaws along the chamber wall; swimming was defined as movement throughout the chamber, and immobility indicated no additional activity beyond what was required to remain afloat. The entire FST procedure was repeated 23 days after PSI administration.

### Tissue and serum collection

Animals were sacrificed 24 h after the last behavioral procedure (i.e., FST 2). Brains were removed, and the prefrontal cortex was dissected with a razor blade and rapidly frozen in a heptane/dry ice mixture, then stored at -80 °C until preparation. Concurrently, trunk blood was collected and allowed to clot in separation tubes at ambient temperature for 30 min before centrifugation at 1500 g for 10 min to separate serum. Collected serum was stored at -80 °C until measurement.

### Quantitative RT-PCR (qRT-PCR)

RNA isolation from tissue was performed using the RNeasy Plus Mini kit (Qiagen Inc.), with total RNA concentration and quality assessed using a NanoDrop ND-1000 Spectrometer (ThermoScientific Inc.). RNA was reverse-transcribed to cDNA using a High-Capacity cDNA Reverse Transcription Kit (Applied Biosystems, USA) with random hexamers. Real-time PCRs were conducted in a CFX96 Touch Real-Time PCR Detection System (Bio-Rad Laboratories, Inc., USA) with CFX Manager software (Bio-Rad, USA). SYBR-based real-time PCR amplification was performed in a total reaction volume of 14 µl, consisting of 7 µl Maxima SYBR Green/ROX qPCR Master Mix (Thermo Fisher Scientific), 0.2 µM forward primer, 0.2 µM reverse primer, and 1.4 µl cDNA template (approximately 70 ng reverse-transcribed total RNA per well). The sequences of the primers used for real-time polymerase amplification can be found in Table 1. The thermal cycling profile consisted of an initial incubation at 95 °C for 10 min, followed by 40 cycles of denaturation at 95 °C for 15 s, annealing at 60 °C for 30 s, and extension at 72 °C for 30 s. A melting curve analysis was performed to confirm the amplification specificity of the PCR products. TaqMan-based real-time PCR amplification was performed with gene-specific primers and probes from the TaqMan Gene Expression Assays (Applied Biosystems), including Rn00571208_g1 for *Arc*, Rn00709588_m1 for *Gria1*, Rn00568514_m1 for *Gria2*, Rn00571479_m1 for *Psd95*, Rn00572130_m1 for *Ntrk1*; Rn00570389_m1 for *Ntrk3*; Rn00579280_m1 for *Ntf3*; Rn00566076_s1 for *Ntf4*; Rn01647301_m1 for *St8sia2*, Rn01427310_m1 for *St8sia4*, and glyceraldehyde 3-phosphate dehydrogenase (*Gapdh*, Mm99999915_g1) as the reference gene. The amplification using the TaqMan Gene Expression PCR Master Mix (Applied Biosystems) was conducted in a total reaction volume of 10 µl, consisting of 5 µl TaqMan Gene Expression PCR Master Mix (Thermo Fisher Scientific), 0.5 µl specific assay, and 1.4 µl cDNA template under the following conditions: 50 °C for 2 min and 95 °C for 10 min, followed by 40 cycles of 95 °C for 15 s and 60 °C for 1 min. The results were calculated using qbasePLUS 2.0 software (Biogazelle) and the ΔΔCt method. The results were normalized to the means of reference genes.

**Table 1:** The sequences of the primers used for real-time polymerase amplification

Gene Name	Forward	Reverse
Bdnf	5’- GGAAATCTCCTGAGCCGAGC − 3’	5’- AGCTTTCTCAACGCCTGTCA − 3’
Ngf	5’- ATCGCTCTCCTTCACAGAG-3’	5’- CATTACGCTATGCACCTCAG-3’
Ntrk2	5’- CCACGGATGTTGCTGACCAAAG-3’	5’- GCCAAACTTGGAATGTCTCGCC-3’
Ngfr	5’- GGAGAGAAACTGCACAGCGACA-3’	5’- CAGGCTACTGTAGAGGTTGCCA-3’
Ppia	5’- TGACTTCACACGCCATAAT-3’	5’- AGATGCCAGGACCTGTATGC-3’

### Western blot analysis

Protein concentration was determined using Bradford Reagent (Sigma‒Aldrich, Saint Louis, MO, USA) according to the manufacturer’s protocol. Equal concentrations of proteins were mixed with 4X Bolt^®^ LDS Sample Buffer (Invitrogen, Waltham, MA, USA) and 10X Bolt^®^ Sample Reducing Agent (Invitrogen) and then denatured at 70 °C for 10 min. Samples were separated on Bolt™ 4–12% Bis–Tris Plus Gels (Invitrogen) under reducing conditions in 20X Bolt^®^ MES SDS Running Buffer (Invitrogen), incubated in 20% ethanol for 10 min, and transferred to immunoblot nitrocellulose membranes (iBlot^®^ 2 Transfer Stacks, nitrocellulose, Invitrogen, Waltham, MA, USA) following the manufacturer’s protocol. Primary antibody for ARC (rabbit, #16290-1-AP, Proteintech, 1:1000) or β-actin (mouse, #A5441, Sigma-Aldrich, 1:20 000) was suspended in the iBind™ Solution Kit and incubated with the membrane at 4 °C overnight or 3 h at room temperature. Following day, secondary antibody anti-rabbit (#ab6721, Abcam, 1:5000) or anti-mouse (#A9044, Sigma‒Aldrich,1:20000) was suspended in the iBind™ Solution Kit and incubated with the membrane for one hour at room temperature. The electrophoretic bands were detected using the Clarity™ Western ECL Substrate (Bio-Rad, Hercules, CA, USA) and FUJIFILM LAS-4000 (Fujifilm Life Science, USA) device. Blot analysis was performed using ImageJ 1.53e software (Wayne Rusband and NIH, USA).

### Enzyme-linked immunosorbent assay (ELISA)

The levels of plasma BDNF were determined using commercially available assay kits optimized for small volumes, following the manufacturer’s instructions. The detection limit for BDNF was 12 pg/mL (ERBDNF, Invitrogen, Carlsbad, CA, USA). Briefly, 100 µL of standards and diluted serum samples (dilution factor: 2×) were added to wells and incubated at room temperature for 2 h with gentle shaking. Assay diluent served as a zero standard for background subtraction to construct a standard curve. After discarding the solution, the wells were washed four times with 400 µL of wash buffer before adding 100 µL of BDNF biotinylated antibody and incubating for 1 h at room temperature. Wells were washed 4 times with buffer, followed by adding 100 µL HRP-avidin to each well for 1 h at 37 °C. Next, 100 µL of TMB substrate was added to each well for 30 min before adding the stop solution. The wells were protected from light at all times after the addition of the substrate solution. The optical density of each well was measured using an automated microplate reader (Multiscan Spectrum, USA) set to 450 nm (correction wavelength set to 550 nm) within 30 min of adding the stop solution. A standard curve was constructed by plotting the mean absorbance for each standard against the concentration to draw a best-fit curve through the points on the graph. The concentration read from the standard curve was then multiplied by the dilution factor. Each sample was measured in duplicate, and the average level from the same rat was used for comparison.

### Statistics

All data were assessed for normality and are presented as the mean ± S.E.M. Repeated measures ANOVAs with time as the within-subject factor and treatment (VEH vs. PSI) and strain (Wistar vs. Wistar Kyoto rats) as the between-subject factors were employed for statistical analysis of the main parameters from all behavioral tests. Additionally, post hoc pairwise comparison tests were used to identify significant differences between groups. For analyses of biochemical data, two-way ANOVA was used for gene expression measurements in the prefrontal cortex, weight gain analysis, and ELISA data. Subsequently, Tukey’s multiple comparison tests were applied to identify significant differences between groups. Statistical analyses were performed using jamovi, v. 2.3.28 (R Core Team 2021; Singmann 2018; Lenth 2020) and GraphPad Prism software (v. 9.1.2). The significance level was set at *p* < 0.05.

## Results

### Novel object recognition

**Fig. 2:**
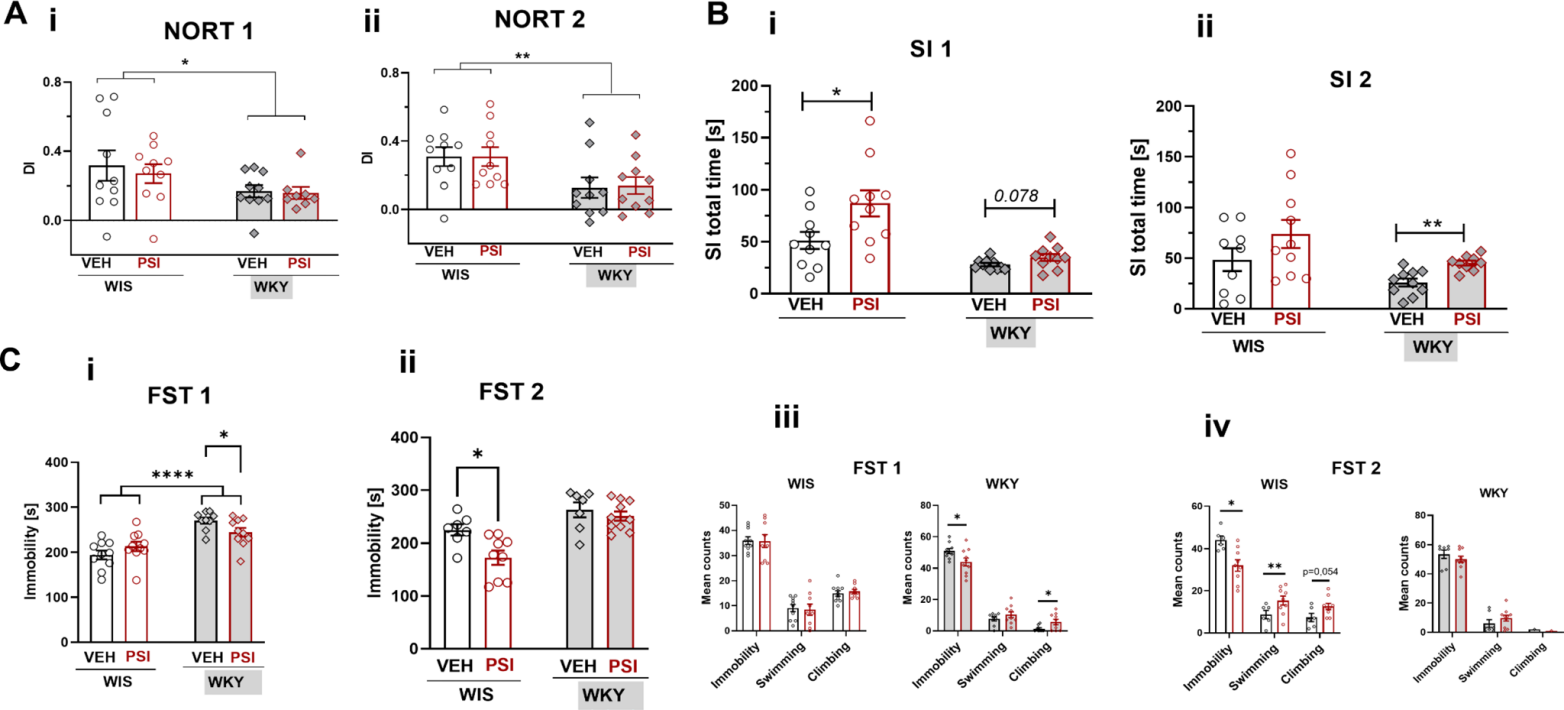
(**A**) Discrimination Index (DI) in the novel object recognition task (NORT). The acquisition trial (T1) in NORT 1 was performed 4 h after PSI administration (A), and in NORT 2 it was performed 6 days after PSI administration. The retention trials (T2) was conducted 24 h following T1. DI was defined as the difference in exploration time between the novel object and the familiar one in T2, divided by the total exploration time. The DI was calculated for all groups, and data are presented as the mean ± S.E.M., *n* = 9–10 rats per group. WKY rats are depicted as rhomboid symbols and grey shaded bars, while WIS rats are represented as squared symbols and white bars. According to ANOVA and subsequent post hoc pairwise comparisons, a significant inter-strain difference in DI between WIS and WKY rats was observed, *p < 0.05; **p < 0.01. (**B**) Outcome measures of the Social Interaction (SI) tests. The SI test was performed 2 days (A) and 8 days (B) after PSI administration. During both SI tests, rats were assessed with a novel social partner for 5 min in the open field. SI total time summarizes all data from sniffing, anogenital sniffing, grooming, following, climbing, and crawling behaviors. Data are presented as mean ± SEM, *n* = 9–10 per group. WKY rats are depicted as rhomboid symbols and grey shaded bars, while WIS rats are represented as squared symbols and white bars. According to repeated measures ANOVA and post hoc pairwise comparisons, in SI 1 test, there was a statistically significant increase in SI time after PSI treatment in WIS rats. In SI 2 test, the increase in social interaction time reached the level of significance in WKY rats, **p < 0.01. Times spent for specific types of behavior are presented in detail in Figure S2 in the supplementary material. (**C**) Outcomes of the Forced Swim Test (FST) performed 9 days after PSI administration (A, C) and 23 days after PSI administration (B, D). WKY rats are depicted as rhomboid symbols and grey shaded bars, while WIS rats are represented as squared symbols and white bars. According to repeated measures ANOVA and post hoc pairwise comparisons, there was a significant inter-strain difference in immobility time in FST 1 between WIS and WKY rats (****p < 0.0001). Additionally, in FST 1 in WKY rats, there was a decrease in immobility after PSI administration. This decrease in immobility occurred concomitantly with an increase in climbing (C) (*p < 0.05 vs. the vehicle group). In FST 2, there was a significant decrease in immobility time after PSI administration in WIS rats (B). This decrease in immobility occurred together with increases in swimming and climbing (D) (*p < 0.05, **p < 0.01 vs. the vehicle group). Data are presented as the mean ± SEM (*n* = 7–10 per group)

The first NOR test (NORT 1) was conducted 4 h after PSI treatment, and the second NOR test (NORT 2) was carried out 6 days post-treatment. Data from both NORTs were analyzed using repeated measures ANOVA, with time (NORT 1 vs. NORT 2) as a within-subject factor, and strain and treatment as between-subject factors. Regarding the Discrimination Index (DI), there was a significant effect of strain (F(1, 34) = 12.68; *p* = 0.001), with DI being lower in WKY rats compared to WIS rats (Fig. 2A (i and ii)). However, time, treatment, and interactions between factors were nonsignificant.

Parameters measured in NORTs, i.e., exploration times of novel and familiar objects in retention trials, total exploration, and distance traveled, are presented in Supplementary Figure S1, panels A, B, and C, respectively. The detailed results of the statistical analyses for these parameters are included in the legends of the Supplementary Figures.

The distance traveled by rats and total exploration time were analyzed using repeated measures ANOVA with two levels of repeated measures factors: phase (phase T1 vs. phase T2) and time (NORT 1 vs. NORT 2). The distance traveled by WKY and WIS rats differed significantly (main effect of strain: F(1,34) = 24.82; *p* < 0.001), with WKY rats traveling a shorter distance than WIS rats in each phase (Supplementary Figure S1, panel C). Within-subject effects were significant for phase (F(1,34) = 27.20; *p* < 0.001), but not for the time factor. Additionally, there was no statistically significant effect of drug or any significant interactions with drug treatment in any case. Although the distance traveled by both strains differed, total exploration time remained unchanged (Supplementary Figure S1, panel B) between strains and was independent of drug treatment.

### Social interaction behavior

The first social interaction test (SI 1) was conducted 2 days after PSI treatment, and the second SI test (SI 2) was carried out 9 days post-treatment. Various types of social behaviors were analyzed using repeated measures ANOVA with two between-subject factors (treatment and strain) and two within-subject factors: behavior type and time (SI 1 vs. SI 2). Total time of social interactions revealed significant effects of strain (F(1,34) = 16.36; *p* < 0.001) and drug treatment (F(1,34) = 8.95; *p* = 0.005), while the effects of time and any interactions were nonsignificant. Specifically, WKY rats exhibited lower interactions with conspecifics compared to WIS rats, and PSI increased the time of social interactions in both strains. However, inter-group differences did not always reach the level of statistical significance in post hoc comparisons (Fig. 2B (i)). According to post hoc pairwise comparisons, in WIS rats, there was a significant difference in social interaction time between the vehicle- and PSI-treated groups in the first SI test (SI 1, *p* = 0.029), but not in the second SI test (SI 2, *p* = 0.181) (Fig. 2B (i) and (ii)). In WKY rats, the difference between vehicle- and PSI-treated groups did not reach the level of statistical significance in the first SI test (SI 1, *p* = 0.078), but it was significant in the second SI (SI 2, *p* < 0.001) (Fig. 2B (i) and (ii)). Regarding specific types of social behaviors (i.e., sniffing, anogenital sniffing, grooming, following, climbing, and crawling), post hoc comparisons revealed that WIS rats showed an increase in anogenital sniffing and following behavior after PSI treatment in the SI 1 test (*p* < 0.001 and *p* = 0.012, respectively) (Supplementary Figure S2 (i)). In WKY rats, there was an increase in anogenital sniffing and following behavior (*p* < 0.001 and *p* = 0.005, respectively) after PSI treatment in the SI 2 (Supplementary Figure S2 (ii)).

### Forced swimming test

The first FST (FST 1) was conducted 9 days after PSI treatment, and the second FST (FST 2) was carried out 23 days post-treatment. Data from both FSTs were analyzed using repeated measures ANOVA, with time (FST 1 vs. FST 2) as a within-subject factor, and strain and treatment as between-subject factors. Considering immobility time, there was a significant effect of strain (F(1,28) = 43.88; *p* < 0.001) and drug (F(1,28) = 5.54; *p* = 0.026). The within-subject effect of time was nonsignificant (*p* = 0.702); however, there were significant interactions time x drug (F(1,28) = 6.84; *p* = 0.014) and time x strain x drug (F(1,28) = 6.45; *p* < 0.017). Immobility time in WKY rats was significantly longer than in WIS rats in both FSTs (*p* < 0.001). Post hoc pairwise comparisons revealed differences in immobility time between VEH- and PSI-treated WKY rats in FST 1 (*p* = 0.0495) (Fig. 2C (i)). Conversely, in FST 2, there was a significant reduction in immobility time in WIS rats after PSI treatment (*p* = 0.012), but not in WKY rats (Fig. 2C (ii)).

When considering 5-second time intervals (Fig. 2C (iii) and (iv)), analysis was performed using repeated measures ANOVA with two between-subject factors (treatment and strain) and time as a within-subject factor (FST 1 vs. FST 2). In immobility mean counts, there was a significant effect of strain (F(1,30) = 19.90; *p* < 0.001) and time (F(1,30) = 9.37; *p* = 0.005), as well as a significant interaction time x strain x and drug (F(1,30) = 5.59; *p* = 0.025). Post hoc comparisons based on the time factor revealed differences in immobility mean counts between FST 1 and FST 2. Pairwise comparisons indicated a decrease in immobility in WKY rats after PSI treatment in FST 1 (*p* = 0.042) and a decrease in immobility in WIS rats after PSI treatment in FST 2 (*p* = 0.007). In swimming mean counts, there was a significant interaction effect time x strain (F(1,30) = 7.05; *p* = 0.013). Pairwise comparisons revealed an increase in swimming in WIS rats, but only in FST 2 (*p* = 0.049). In climbing mean counts, there was a significant effect of strain (F(1,30) = 66.48; *p* < 0.001) and significant within-subject effects of time (F(1,30) = 34.71; *p* < 0.001) and the interaction time x strain x drug (F(1,30) = 10.64; *p* = 0.003). WKY rats exhibited very little climbing behavior compared to WIS rats (*p* < 0.001); however, multiple pairwise comparisons revealed an increase in climbing after PSI treatment in WKY rats in FST 1 (*p* = 0.030), accompanied by a decrease in the immobility period, as mentioned above. In summary, statistical analyses revealed differences in immobility and climbing after PSI treatment in WKY rats, but only in FST 1. Conversely, pronounced changes in activity after PSI treatment were observed in WIS rats, but not in WKY rats, during FST 2. For a detailed summary of behavioral changes, see Table 2. 

**Table 2:** Summary of behavioral changes observed in each parameter or test, considering both the strain (WKY and WIS) and treatment (PSI and VEH). The arrows (↑ and ↓) indicate whether there was an increase or decrease in each parameter or test, and “No significant change, ns” means that there were no significant differences observed

Test	Parameter	Change in WKY (PSI vs. VEH)	Change in WIS (PSI vs. VEH)
NORT 1	Discrimination Index	ns	ns
	Distance Traveled	ns	ns
	Total Exploration Time	ns	ns
NORT 2	Discrimination Index	ns	ns
	Distance Traveled	ns	ns
	Total Exploration Time	ns	ns
SI 1	Social Interaction	ns	(↑)
	Anogenital Sniffing	ns	(↑)
	Following	ns	(↑)
SI 2	Social Interaction	(↑)	ns
	Anogenital Sniffing	(↑)	ns
	Following	(↑)	ns
FST 1	Immobility Time	(↓)	ns
	Climbing	(↑)	ns
FST 2	Immobility Time	ns	(↓)
	Swimming	ns	(↑)
Weight gain	Weight Gain	ns	(↓)

### Weight gain

Weight gain significantly differed among the studied groups of rats a 3 weeks after PSI administration. Specifically, there was a significant effect of treatment (F(1,36) = 7.221; *p* = 0.0108). PSI-treated WIS rats gained significantly less weight than their controls (Fig. 1B), while in WKY rats there was no difference between PSI-treated group and control group.

### Gene expression of neuroplasticity-related genes in the PCX

**Fig. 3:**
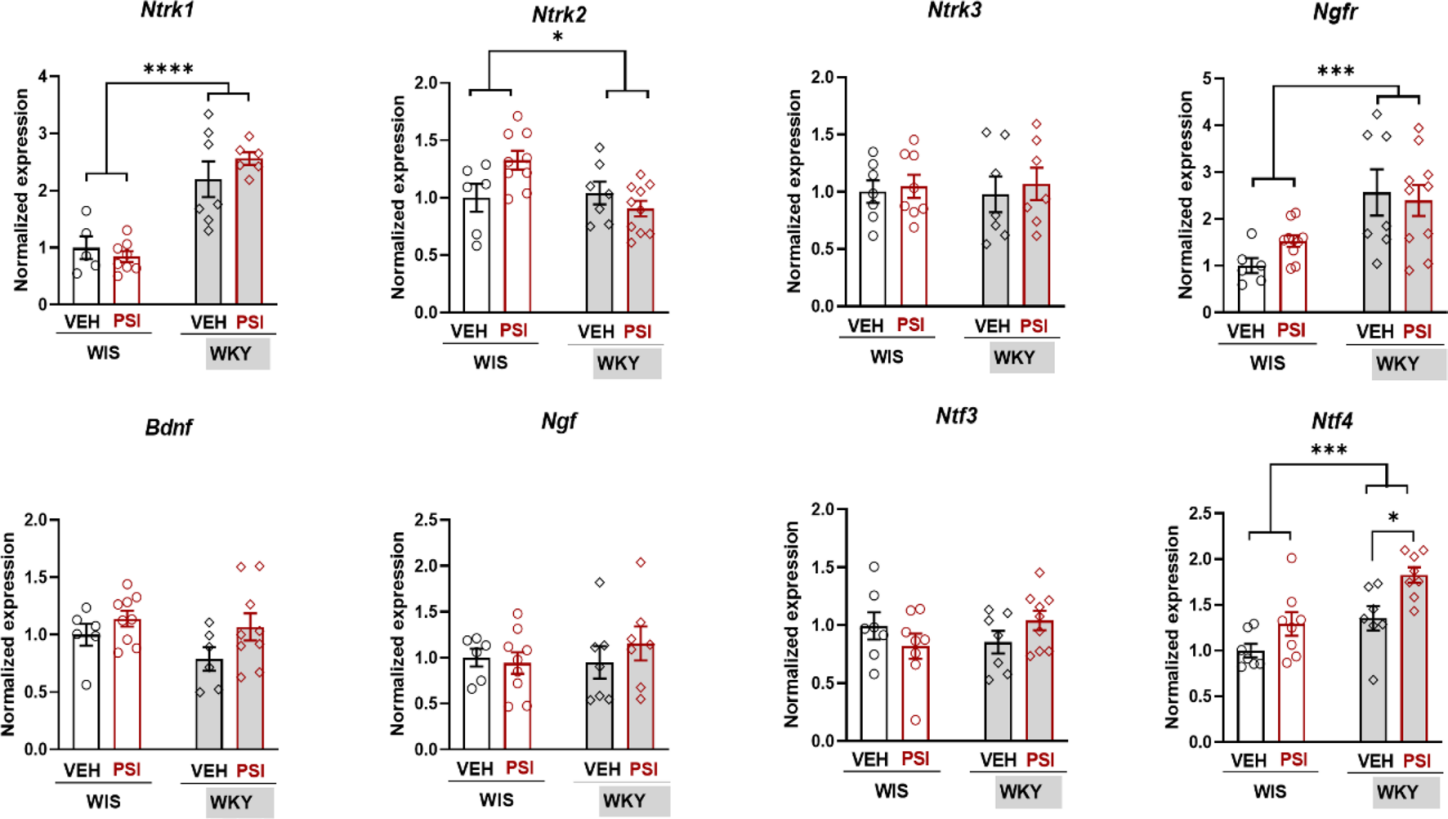
Fold changes in the mRNA expression of neurotrophins and their receptors. Gene expression was measured in the prefrontal cortex. Individuals are represented in all the graphs with *n* = 7–10 per group. WKY rats are depicted as rhomboid symbols and grey shaded bars, while WIS rats are represented as squared symbols and white bars. Groups treated with psilocybin (PSI) are shown in red. Statistical analyses were performed with two-way ANOVA with Tukey multiple comparisons post hoc test. Statistically significant changes between groups are indicated as *p < 0.05, **p < 0.01, and ****p < 0.0001; Abbreviations: Ntrk1—neurotrophic receptor tyrosine kinase 1; Ntrk2—neurotrophic receptor tyrosine kinase 2; Ntrk3—neurotrophic receptor tyrosine kinase 3; Ngfr—nerve growth factor receptor; Bdnf—brain-derived neurotrophic factor; Ngf—nerve growth factor; Ntf3—neurotrophin 3; Ntf4—neurotrophin 4

**Fig. 4:**
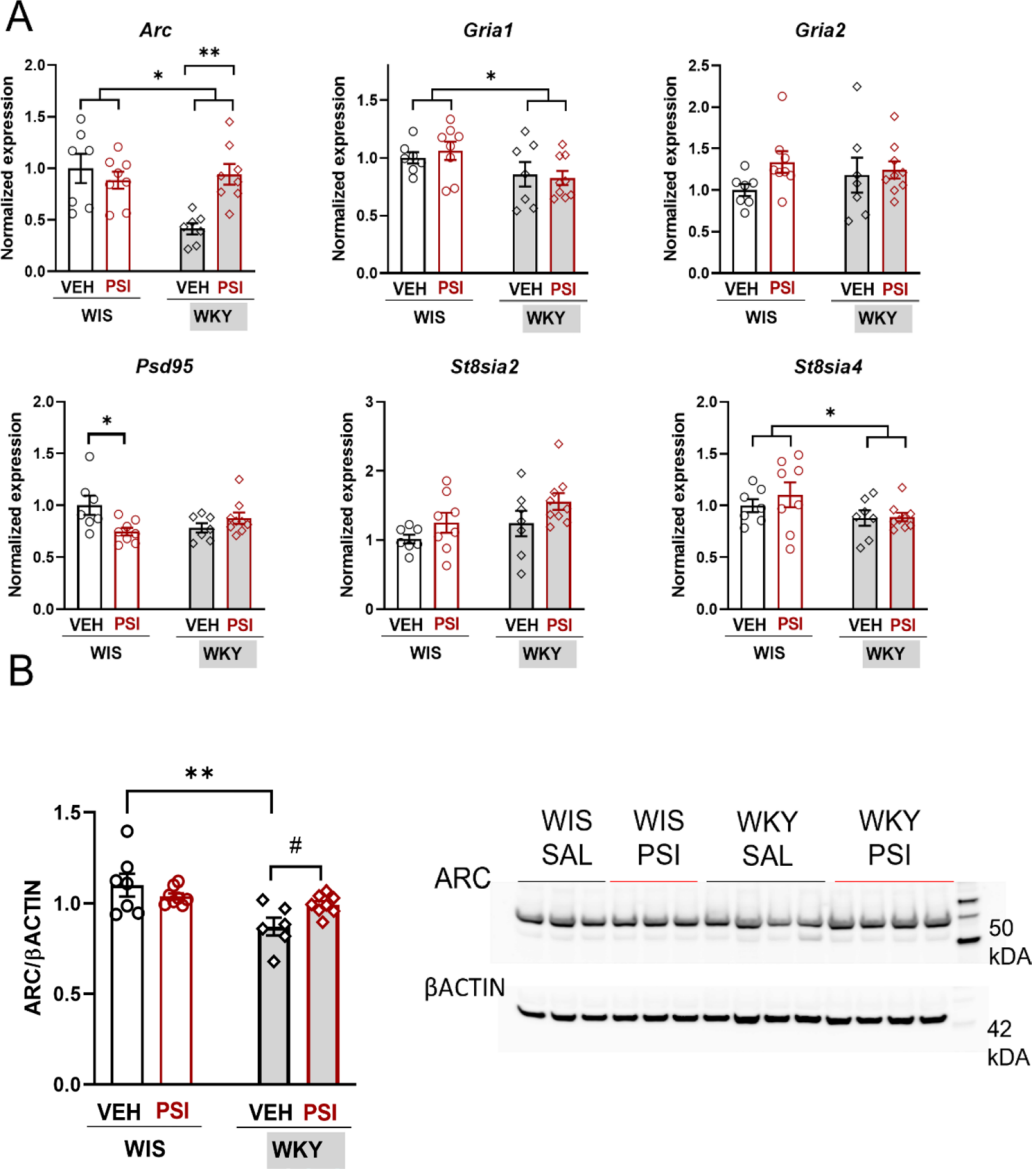
(**A**) Fold changes in the mRNA expression of selected genes involved in synaptic plasticity. Gene expression was measured in the prefrontal cortex. Individuals are shown in all the graphs with *n* = 7–10 per group. WKY rats are presented as rhomboid symbols and grey shaded bars, while WIS rats are represented as squared symbols and white bars. Groups treated with psilocybin (PSI) are shown in red. Statistical analyses were performed with two-way ANOVA with Tukey multiple comparisons post hoc test. Statistically significant changes between groups are indicated as *p < 0.05 and **p < 0.01; Abbreviations: Arc—activity-regulated cytoskeletal-associated protein; Gria1—glutamate receptor, ionotropic, AMPA1 (alpha 1); Gria2—glutamate receptor, ionotropic, AMPA2 (alpha 2); Psd95—postsynaptic density protein 95; St8sia2—ST8 alpha-N-acetyl-neuraminide alpha-2,8-sialyltransferase 2; St8sia4—ST8 alpha-N-acetyl-neuraminide alpha-2,8-sialyltransferase 4. **B)** The normalized expression of ARC protein in the prefrontal cortex was measured by Western blot analysis. Individuals are shown in all the graphs with *n* = 7–10 per group. WKY rats are presented as rhomboid symbols and grey shaded bars, while WIS rats are represented as squared symbols and white bars. Groups treated with psilocybin (PSI) are shown in red. Statistical analyses were performed with two-way ANOVA with Tukey multiple comparisons post hoc test. Statistically significant changes between groups are indicated as **p < 0.01 and #*p* < 0.05. Representative Western blot image for ARC in PCX

The statistical analysis of gene expression values was performed using a two-way ANOVA with two factors: Strain (WKY vs. WIS rats) and Treatment (PSI vs. VEH), followed by Tukey’s multiple comparisons tests. The results are presented in Fig. 3 (*Bdnf* and related neurotrophins *Ntf3/Ntf4* and its receptors) and Fig. 4A (other neuroplasticity-related genes). Expression of certain genes in WKY strain significantly differed from WIS strain (main effect of strain): *Arc* (strain factor F(1, 26) = 7.095; *p* = 0.0131), *Gria1* (strain factor F(1, 27) = 6.134; *p* = 0.0198), *St8sia4* (strain factor F(1, 27) = 4.438; *p* = 0.0446), *Ntf4* (strain factor F(1, 26) = 16.50; *p* = 0.0004), *Ntrk1* (strain factor F(1, 23) = 30.01; *p* < 0.0001), *Ntrk2* (strain factor F(1, 28) = 4.563; *p* = 0.0416), and *Ngfr* (strain factor F(1, 29) = 15.05; *p* = 0.0006). Generally, the expression of *Arc*, *Gria1*, and *St8sia4* was lower in WKY than in WIS rats, while the expression of *Ntrk1*, *Ngfr*, and *Ntf4* was higher in WKY than in WIS rats.

Considering the effect of treatment, PSI treatment influenced the expression of *Arc* (treatment factor F(1, 26) = 4.347; *p* = 0.0470), *Ntf4* (treatment factor F(1, 26) = 16.50; *p* = 0.0004), and *Bdnf* (treatment factor F(1, 26) = 4.249; *p* = 0.0494). Additionally, an interaction between strain and treatment factors occurred in the case of *Arc* (F(1, 26) = 10.57; *p* = 0.0032), *Psd95* (F(1, 27) = 8.477; *p* = 0.0071), and *Ntrk2* (F(1, 28) = 6.723; *p* = 0.0150). Tukey’s multiple comparisons test revealed that PSI induced the expression of two genes compared to VEH in WKY rats, i.e., *Arc* (*p* = 0.0044) and *Ntf4* (*p* = 0.0247). On the other hand, PSI treatment caused the decrease in the expression of *Psd95* in WIS rats (*p* = 0.0297).

### Changes in ARC protein in the PCX

Expression of ARC protein in WKY strain significantly differed from WIS strain (main effect of strain: F(1, 25) = 12.35; *p* = 0.017). Additionally, an interaction between strain and treatment factors occurred in the case of ARC (F(1, 25) = 5.483; *p* = 0.0275). Tukey’s multiple comparisons test revealed that PSI induced the expression of ARC protein compared to VEH in WKY rats (*p* = 0.0260); Fig. 4B.

### Serum level of BDNF

No significant changes were observed in the serum level of Brain-Derived Neurotrophic Factor (BDNF) between WIS and WKY rats, as well as after PSI treatment (Supplementary Figure S2).  For a summary of biochemical changes, see Table 3.

**Table 3:** Summary of biochemical changes observed in each parameter, considering both the strain (WKY and WIS) and treatment (PSI and VEH). “No significant change, ns” means that there were no significant differences observed

Target	Strain effect	PSI effect
*Arc*	Lower in WKY (↓)	Increase with PSI in WKY (↑)
*Gria1*	Lower in WKY (↓)	ns
*St8sia4*	Lower in WKY (↓)	ns
*Ntf4*	Higher in WKY (↑)	ns
*Ntrk1*	Higher in WKY (↑)	ns
*Ntrk2*	Lower in WKY (↓)	ns
*Ngfr*	Higher in WKY (↑)	ns
*Psd95*	ns	Decrease with PSI in WIS (↓)
*Bdnf*	Lower in WKY (↓)	Increase with PSI in WKY (↑)
BDNF serum level	ns	ns
ARC protein level	Lower in WKY (↓)	Increase with PSI in WKY (↑)

## Discussion

WKY rats have been established as a model for TRD, as supported by previous studies (Aleksandrova et al., 2019; Papp et al. 2018). In our investigation of the effects of PSI in these rats, we not only gained insights into PSI’s actions but also revealed valuable information about the WKY strain itself. This information holds potential significance in enhancing our comprehension of the TRD model.

### Strain variations and behavioral disparities in object recognition and coping strategies

Our findings reveal significant behavioral differences between WIS and WKY rats during the NORT, primarily attributable to inherent strain characteristics rather than the effects of PSI treatment. WKY rats demonstrated a consistently lower Discrimination Index (DI) in both the immediate (NORT 1) and delayed (NORT 2) phases of testing, suggesting impaired cognitive functions or memory recall compared to WIS rats. This impaired recognition ability in WKY rats suggests cognitive deficits, potentially reflecting their anxiogenic phenotype and increased reliance on passive coping strategies, which is evident in their preference for familiar objects over novel ones (Antunes and Biala 2012). Moreover, despite differences in DI, the total exploration time remained consistent between the two rat strains in both NORT phases, indicating that the observed behavioral disparities primarily relate to cognitive aspects rather than overall activity levels. However, WKY rats traveled shorter distances during these tests, highlighting a behavioral pattern that may correspond with their less active and more passive coping strategies.

### Psilocybin’s influence on cognitive behaviors

Contrary to expectations and previous research suggesting the cognitive-enhancing effects of psychedelics, PSI treatment did not significantly influence the results of either NORT 1 or NORT 2 in our study. This finding is consistent with recent studies which have reported variable effects of psychedelics on cognitive functions and exploratory behaviors. For instance, Buzzelli et al. (2023) demonstrated that while PSI microdoses normalized short-term object recognition deficits in Fmr1-Δexon 8 rats, a model for Fragile X syndrome, they did not enhance cognitive performance in wild-type Sprague-Dawley rats. Cini et al. (2019) observed that a single dose of LSD increased exploratory time for novel objects in an age-dependent manner, while Wojtas et al. (2021) found no significant differences in novel object preference shortly after treatment. Additionally, de la Fuente Revenga et al. (2021) and Thakur et al. (2022) reported that both DOI and PSI did not alter NORT behaviors in mice 24 h post-administration, supporting our findings that PSI’s influence on recognition memory may be subtle and context-dependent (Rambousek et al. 2014).

### Social behavior variations between strains

The analysis of the Social Interaction Test (SI) highlighted pronounced strain-based differences in social behaviors between WIS and WKY rats. Throughout both assessments, SI 1 and SI 2, WIS rats exhibited markedly more social interaction than their WKY counterparts. These interactions were particularly characterized by increased anogenital sniffing and following behavior, suggesting a naturally higher social propensity in WIS rats, as supported by prior studies (Nam et al. 2014). WKY rats are distinct for their behavioral inhibition, often displaying reduced activity and passivity in social contexts (Paré et al., 2000) and other stressful situations (Paré 1994). This strain also shows diminished locomotor activity (Ferguson et al., 2004), an increased susceptibility to stress-induced ailments (Pare, 1989), heightened stress responses, and impaired learning and memory processes (Ferguson et al., 2004). Additionally, WKY rats demonstrate significant avoidance behavior, a critical element in the pathology of anxiety disorders, more prominently than outbred Sprague Dawley rats and within their own strain across sexes (Servatius et al. 2008; Nam et al. 2014).

### Impact of psilocybin on social interactions

Turning to the effects of PSI, our findings contribute to the understanding of how this psychedelic substance influences social behaviors. During the first Social Interaction test (SI 1), PSI treatment significantly enhanced social behaviors in WIS rats compared to the vehicle-treated controls, specifically increasing anogenital sniffing and following behavior. This suggests a pro-social effect of PSI in WIS rats, enhancing their social engagement and curiosity toward conspecifics. However, in SI 2, while the strain-based differences remained prominent, the pro-social effects of PSI did not significantly alter the social interaction patterns observed in WIS rats from the first test, though significant behavioral modifications were evident in WKY rats.

These observations are crucial in understanding the differential responses to psychedelics between these two rat strains. The immediate and noticeable response of WIS rats to PSI treatment contrasts with the more subtle changes in WKY rats, indicating that WKY rats may require a longer duration to manifest the pro-social benefits of PSI. This delay could be pivotal in devising therapeutic strategies involving psychedelics, particularly in the context of TRD.

Historically, the acute effects of psychedelics on social behaviors were primarily analyzed through aggression-related tests in rodents, where substances like DMT and psilocin were noted to reduce aggression, unlike LSD (Uyeno 1967; Sbordone et al. 1979; Walters et al. 1978). Our study extends these findings by exploring the nuanced impacts of PSI on non-aggressive social interactions, providing a fresh perspective on the potential of psychedelics to modulate social behavior in a therapeutic setting.

### Variable responses to psilocybin in the forced swim test (FST)

The Forced Swim Test (FST) is a widely used method for screening antidepressants, where behavioral immobility in animals is interpreted as a response to inescapable stress, traditionally viewed as a model of depressive behavior (Porsolt et al. 1977). However, the FST has faced increasing scrutiny and criticism. Critics argue that immobility might not necessarily indicate despair or depression but could instead represent a strategic, adaptive behavior aimed at conserving energy in a stressful situation (Castagné et al. 2011; Molendijk and de Kloet 2015). This interpretation suggests that the FST may actually assess coping strategies rather than depressive states. Furthermore, although the FST has been critical in characterizing the WKY rat strain as exhibiting depressive-like behaviors due to their increased immobility (Paré 1994), recent critiques have questioned the direct correlation of FST performance with depressive behavior. It is argued that the immobility observed might reflect broader psychobiological strategies rather than specific depressive symptoms (Commons et al. 2017; Campus et al. 2015). These criticisms highlight the need for cautious interpretation of FST results and advocate for its use alongside other behavioral assays to obtain a more holistic understanding of an animal’s behavioral profile and the potential antidepressant effects of treatments.

Despite these criticisms, the FST remains a valuable tool for initial screening of antidepressant activity, especially when combined with other methods that assess different aspects of behavior and neurobiology. In our study, the FST was employed to discern the antidepressant effects of PSI on different rat strains, allowing us to observe strain-specific differences in response to PSI.

Our findings align with the broader literature indicating inconsistent behavioral outcomes of PSI in the FST, which may be influenced by factors like the timing of assessment and the genetic background of the animals tested (Hibicke et al. 2020; Risca et al., 2021; Jones et al. 2020; Hesselgrave et al. 2021; Jefsen et al. 2019). Notably, Hibicke et al. (2020) observed a long-lasting antidepressant effect of a single PSI administration in WKY rats, lasting up to five weeks, whereas studies involving microdosing regimens or different strains such as C57Bl6/J mice or Flinder Sensitive Line rats did not demonstrate significant effects (Jefsen et al. 2019; Jones et al. 2020; Risca et al., 2021).

### Strain-specific behavioral outcomes and the effect of PSI

In WKY rats, known for their rapid acquisition of learned helplessness behavior (Paré 1994; Nam et al. 2014), prolonged immobility may also be indicative of enhanced psychomotor retardation. This strain exhibits unique responses in the FST, which may also be influenced by their elevated baseline activity in locus coeruleus (LC) neurons, potentially impacting noradrenergic signaling (Bruzos-Cidón et al. 2014; Scholl et al. 2010). Interestingly, the distinct behavioral outcomes in WKY rats could be attributed to their noradrenergic system’s dynamics, as indicated by altered climbing behavior observed post-PSI administration, a response not prominently seen in Wistar rats until much later (Detke et al. 1995; Lucki 1997).

### Impact of PSI on weight gain

Pharmacological treatments for obesity often target the serotonergic system due to its pivotal role in regulating eating behaviors. Serotonin significantly influences appetite control both through hypothalamic and extrahypothalamic pathways (Voigt and Fink 2015). Medications such as fenfluramine, which directly releases serotonin, and sibutramine, which inhibits serotonin reuptake, have been effective in managing obesity by enhancing serotonergic activity (Burke and Heisler 2015). Additionally, PSI, which acts on serotonin receptors including 5-HT1B, 5-HT2C, and 5-HT6, has been associated with weight modulation. A notable study by Simonsson et al. (2021) reported a reduced prevalence of obesity in individuals using PSI, suggesting a potential influence on weight management, although the underlying mechanisms remain to be elucidated. Our research further explores this connection, demonstrating that a single dose of PSI can significantly reduce body weight in WIS rats. This effect, however, was manifested differentially across strains and over time. Specifically, weight reduction became statistically significant at 21 days post-administration, but was not evident at 7 days, underscoring a delayed response to PSI treatment (unpresented observations). Contrastingly, in WKY rats, no significant weight change was observed, possibly due to inherent metabolic differences between the strains. For instance, while chronic restraint stress induces similar elevations in plasma corticosterone (CORT) levels in WKY and other rat strains, WKY rats exhibit less pronounced body weight changes under the same stress conditions compared to Fischer 344 (F344) rats (Andrus et al., 2021; Jung et al. 2020). Further, quantitative trait locus (QTL) analyses have identified various genetic markers associated with behavioral and metabolic irregularities, including a predisposition to diabetes, which may affect body weight regulation through the hypothalamic-pituitary-adrenal (HPA) axis in WKY rats (Redei et al. 2023). These findings suggest a complex interplay between genetic background, serotonergic modulation, and metabolic traits that influence the efficacy of PSI and other serotonergic drugs in obesity treatment.

### Insights into neuroplasticity and genetic influence

The differential timing and nature of responses to PSI treatment in our study suggest that distinct genetic factors may modulate the effects of PSI in these rat strains. The role of early response genes and mRNA expression patterns observed post-PSI administration indicate a potentially rapid onset of neuroplasticity changes, particularly in WKY rats (Calder and Hasler 2023; Ray 2010). This finding underscores the complexity of the genetic and neurobiological underpinnings influencing the efficacy of PSI and potentially other psychedelics in modulating depressive behaviors and neuroplasticity.

### Changes in the levels of neurotrophins

#### Impact of psilocybin on BDNF mRNA expression

Our two-way analysis highlighted the significant impact of PSI administration on the mRNA expression encoding BDNF in the PFC. However, post hoc analysis did not reveal statistical significance between the control and PSI-administered groups. This could be due to the timing of our assessment—24 days after PSI administration—which may miss the peak expression period of BDNF mRNA. Studies have shown that BDNF mRNA levels can increase within 2 h, but the effect may diminish significantly within 24 h (Ly et al. 2018). Additionally, Martin et al. (2014) demonstrated that administering LSD every other day for one month resulted in a long-term increase in neuroplasticity-related gene expression, including BDNF, in the medial PFC weeks after treatment cessation.

#### Role of NT4 in neuroplasticity and memory

Further to the impact on BDNF, our study found that PSI significantly influenced NT4 expression, a neurotrophin involved in enhancing glutamatergic synaptic transmission and crucial for synaptic activity and long-term memory functions (Lessmann et al. 1994; Wang and Poo 1997). Three weeks post-PSI administration, we observed an increase in NT4 expression, potentially linked to improvements in long-term memory processes. This effect underscores the role of NT4, often in concert with BDNF, in promoting neuronal survival and synapse formation more effectively than BDNF alone (Fan et al. 2000). Both NT4 and BDNF also induce the expression of the immediate-early gene c-fos in cortical neuronal cultures, highlighting their significant contributions to neuronal plasticity.

#### Insights into neuroplasticity

Recent findings suggest that psychedelics like PSI promote neuroplasticity and associated behavioral effects through high-affinity binding to the Trkβ receptor. Psychedelics are not direct Trkβ agonists but act allosterically to enhance the action of endogenous BDNF at active synapses (Moliner et al. 2023). Our study noted significant strain-dependent changes in Ntrk2 gene expression, which encodes the Trkβ receptor, particularly highlighting differential effects between WIS and WKY rats.

#### Differential expression of neurotrophic factors in rat strains

Additionally, our findings revealed increased expression of Ntrk1 and Ngfr in WKY rats, encoding the TrkA and p75 receptors, respectively. This increase, particularly in response to stress, suggests a potential neuroadaptive mechanism allowing for enhanced neuronal survival and functional maintenance under stress (Bothwell, 2016). Although PSI did not alter the expression of these genes, the observed upregulation in WKY rats suggests a strain-specific adaptive response to environmental stressors.

#### The impact on the modulation of synaptic plasticity

Our study revealed that the expression of the Arc gene, which encodes the ARC protein (activity-regulated cytoskeleton-associated protein), showed changes both in a strain factor-dependent manner and after PSI treatment. Previous studies have indicated that psychedelic-induced changes in Arc expression are typically acute, with changes noted shortly after administration (Calder and Hasler, 2023). For example, Benekareddy et al. (2013) observed an increase in Arc levels in cortical tissues two hours after DOI administration, although not in hippocampal tissues. Jefsen et al. (2021) reported that cortical Arc expression was not significantly increased 90 min after a single dose of PSI, though other immediate early genes like c-fos were induced (Jefsen et al. 2021). Additionally, Pei et al. (2004) demonstrated that DOI-induced increases in cortical Arc expression could be blocked by antagonists for 5-HT2A receptors, as well as AMPA and NMDA antagonists. Interestingly, Arc expression in the cortex has been found to overlap with c-fos reactivity in the same population of cortical cells, mainly in neurons expressing AMPA and NMDA receptors but not 5-HT2A receptors (Pei et al. 2004; Maćkowiak et al., 1999). The role of ARC in cognitive flexibility is further highlighted by experiments in Arc knockout (Arc−/−) mice, where short-term learning remains intact, but there is a deficiency in memory consolidation and maintenance, suggesting a crucial role in long-term memory and plasticity (Plath et al. 2006). This insight is aligned with the increased expression of Ntf4, which has been shown to reinforce evoked synaptic transmission and enhance glutamatergic synaptic transmission in cultured hippocampal neurons (Lessmann et al. 1994; Wang and Poo 1997). Furthermore, while we observed a significant decrease in Gria1 expression (encoding AMPA receptor subunit 1, GluR1) in WKY rats, no subsequent increase was noted post-PSI administration, aligning with findings from Jefsen et al. (2021). A recent study by Wojtas et al. (2022) also highlighted that while PSI elevated NMDA receptor subunits like GluN2B, it did not affect AMPA receptor protein levels (GluA1, GluA2) in the rat frontal cortex (Wojtas et al. 2022). These findings underscore the critical role of glutamatergic neurotransmission in neuronal plasticity, particularly through AMPA receptors, which are known to significantly influence LTP processes (Mead and Stephens 2003). However, it’s also noted that hippocampal LTP may involve non-glutamatergic mechanisms, such as atypical retrograde nitric oxide signaling initiated by calcium influx through L-type calcium channels, rather than NMDA receptors on the postsynaptic membrane (Zahid et al. 2017). Moreover, LTP can endure for several hours in vitro or several weeks to months in vivo, highlighting the potential for long-lasting synaptic modifications (Abraham et al. 2002).

The dissociation between Arc and Gria1 expression in our study suggests that distinct molecular mechanisms might underpin the observed cognitive deficits and alterations in synaptic plasticity following PSI treatment. Remarkably, the reversal in mRNA expression of Arc noted 24 days post-administration indicates persistent changes in ARC-related activity, pointing to long-term alterations in synaptic function. These findings prompt further investigation to fully understand the enduring impacts of PSI on neuroplasticity and cognitive processes.

### Limitations

Our experiments were meticulously planned to maximize the information obtained while minimizing the use of laboratory animals, aligning with the 3R principle (Reduce, Refine, Replace), and were exclusively conducted using male subjects. However, we recognize that sex, as a biological factor, plays a key role in study design, analysis and in the process of reporting results (Shore et al. 2024). Prior research by Hesselgrave et al. (2021) has indicated that female mice showed comparable behavioral responses to PSI. The choice to use only male rats in the current study was primarily driven by the goal to characterize the WKY strain under controlled conditions, aiming to eliminate variables that could introduce additional complexity to the behavioral analysis. However, this decision does not account for the full spectrum of biological variability, especially since sex differences could significantly impact the generalizability of our findings. Including female rats in future studies will be crucial to address this limitation, as it will provide a more comprehensive understanding of the WKY strain’s response to PSI and enhance the external validity of our research findings.

In our study, we used 7-week-old rats as they complement our previous research (Korlatowicz et al. 2023a; b). However, it should be emphasized that rats at this stage of development may not fully represent the physiology or behavior of adult individuals, which are usually the focus of such studies. Younger rats undergo significant neurological development, and their neurochemical environment may differ from that of fully mature rats, which can affect their responses to psychoactive substances such as psilocybin.

## Conclusion

Our study explored the effects of PSI on WKY and WIS rats, revealing significant strain-specific responses that enhance our understanding of TRD. We observed distinct behavioral and neuroplastic responses to PSI, particularly in neuroplasticity-related genes like Arc, which showed increased expression in WKY rats more than three weeks post-administration. These findings underscore the potential for lasting brain function modulation induced by PSI, suggesting its utility in long-term TRD treatment strategies. However, the use of only male rats points to a need for broader research incorporating female subjects to fully capture the spectrum of PSI effects and ensure the generalizability of our findings. This study highlights the complexity of PSI’s impact, advocating for personalized approaches in the therapeutic use of psychedelics.

## Electronic supplementary material

Below is the link to the electronic supplementary material.

Supplementary Material 1
