# Correction to: Unraveling psilocybin’s therapeutic potential: behavioral and neuroplasticity insights in Wistar-Kyoto and Wistar male rat models of treatment-resistant depression

**DOI:** 10.1007/s00213-024-06654-1

**Published:** 2024-07-20

**Authors:** Magdalena Kolasa, Agnieszka Nikiforuk, Agata Korlatowicz, Joanna Solich, Agnieszka Potasiewicz, Marta Dziedzicka-Wasylewska, Ryszard Bugno, Adam Hogendorf, Andrzej Bojarski, Agata Faron-Górecka

**Affiliations:** 1 Department of Pharmacology, Maj Institute of Pharmacology Polish Academy of Sciences, Kraków, Poland; 2 Department of Behavioral Neuroscience & Drug Development, Maj Institute of Pharmacology Polish Academy of Sciences, Kraków, Poland; 3 Department of Medicinal Chemistry, Maj Institute of Pharmacology Polish Academy of Sciences, Kraków, Poland

**Correction to: Psychopharmacology**

10.1007/s00213-024-06644-3.

In the published paper, the first names and surnames of most authors were reversed during the publication process. They should be corrected to the following format:

Magdalena Kolasa, Agnieszka Nikiforuk, Agata Korlatowicz, Joanna Solich, Agnieszka Potasiewicz, Marta Dziedzicka-Wasylewska, Ryszard Bugno, Adam Hogendorf, Andrzej Bojarski & Agata Faron-Górecka

