# Supplementary material for: Unraveling psilocybin’s therapeutic potential: behavioral and neuroplasticity insights in Wistar-Kyoto and Wistar male rat models of treatment-resistant depression
Source: Psychopharmacology (Berl). 2024 Jul 4;242(7):1607–25. doi: 10.1007/s00213-024-06644-3 (PMC12226618; doi:10.1007/s00213-024-06644-3)
Supplement: Supplementary file 1 — Supplementary Material 1 [file 213_2024_6644_MOESM1_ESM.docx]

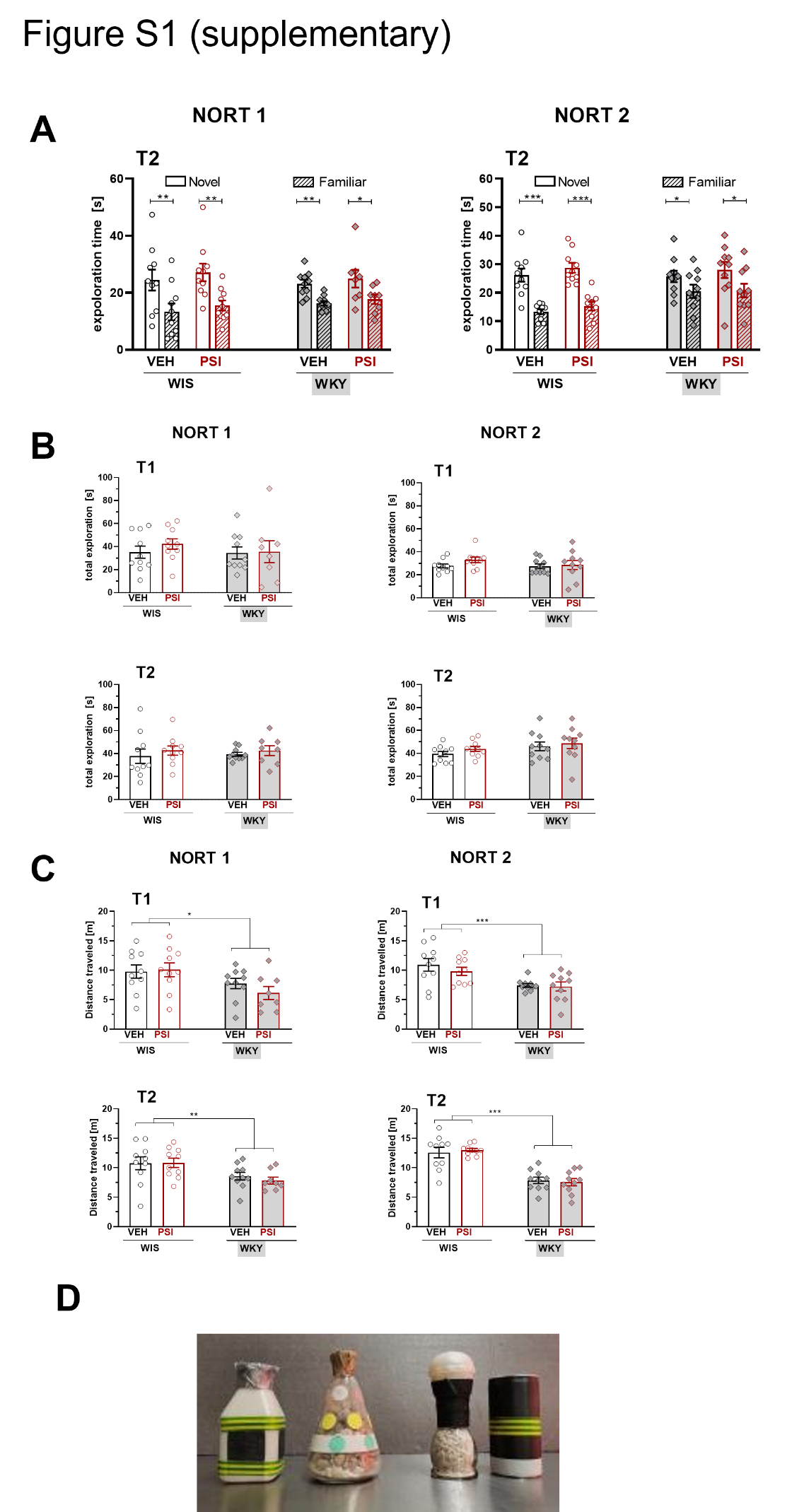


**Figure S1.** Exploration time and locomotor activity in NORT assays. The data are presented as the mean ± SEM of 9–10 rats. WKY rats are depicted as rhomboid symbols and grey shaded bars, while WIS rats are represented as squared symbols and white bars. A) Comparison of exploratory and novel preference during the retention trial (T2, test phase) in NORT 1 and NORT 2. Exploration times of novel and familiar objects were analyzed using repeated measures ANOVA with two levels of repeated measures factors: novelty (novel vs familiar) and time (NORT 1 vs NORT 2). Within-subject effects in ANOVA were significant for novelty (F(1, 34) = 100.71; p < 0.001) and for the interaction novelty x treatment (F(1, 34) = 8.79; p = 0.006). Post hoc analysis revealed differences between exploration of novel and familiar objects in retention trials (T2, test phase) in both NORT 1 and NORT 2, as expected. According to the interaction novelty x treatment, post hoc comparisons revealed a significant overall difference in exploring familiar objects by rats after vehicle and PSI treatment (p = 0.004); however, specific differences between individual groups were not evident. Furthermore, the ANOVA results indicated that the within-subject effect of time was significant (F(1, 34) = 4.53; p = 0.041); however, post hoc comparisons did not reveal any significant differences between individual groups according to this factor. Additionally, there were no significant between-subjects main effects of strain, treatment, or interactions between any other factors, besides those mentioned above. According to post hoc pairwise comparisons, *p < 0.05, **p < 0.01, and ***p < 0.001 for novel vs. familiar object. B) Total exploration time. Comparison of the total time spent with both objects by all groups in specific trials of NORT 1 and NORT 2. The total exploration time was analyzed using repeated measures ANOVA with two levels of repeated measures factors: phase (phase T1 vs phase T2) and time (NORT 1 vs NORT 2). There were some within-subject effects according to phase (T1 vs T2, F(1,34) = 40.03; p < 0.001) and interactions phase x strain (F(1,34) = 5.43; p = 0.026) and phase x time (F(1,34) = 13.66; p < 0.001), indicating some differences in total exploration time between phases (T1 vs T2 in specific NORT), as well as some differences between specific phases (i.e., T1 and T2) between NORTs. However, there were no statistically significant interactions with drug treatment in any case. C) Comparison of locomotor activity in specific trials of NORT 1 and NORT 2. The distance traveled by rats was analyzed using repeated measures ANOVA with two levels of repeated measures factors: phase (phase T1 vs phase T2) and time (NORT 1 vs NORT 2). The distance traveled by WKY and WIS rats differed significantly (main effect of strain: F(1,34) = 24.82; p < 0.001), with WKY rats traveling a shorter distance than WIS rats in each phase (post hoc pairwise comparisons, *p < 0.05, **p < 0.01, and ***p < 0.001). Within-subject effects were significant for phase (F(1,34) = 27.20; p < 0.001), but not for the time factor. Additionally, there was no statistically significant effect of drug or any significant interactions with drug treatment in any case. Although the distance traveled by both strains differed, total exploration time remained unchanged (Supplementary Figure S1, panel B) between strains and was independent of drug treatment.


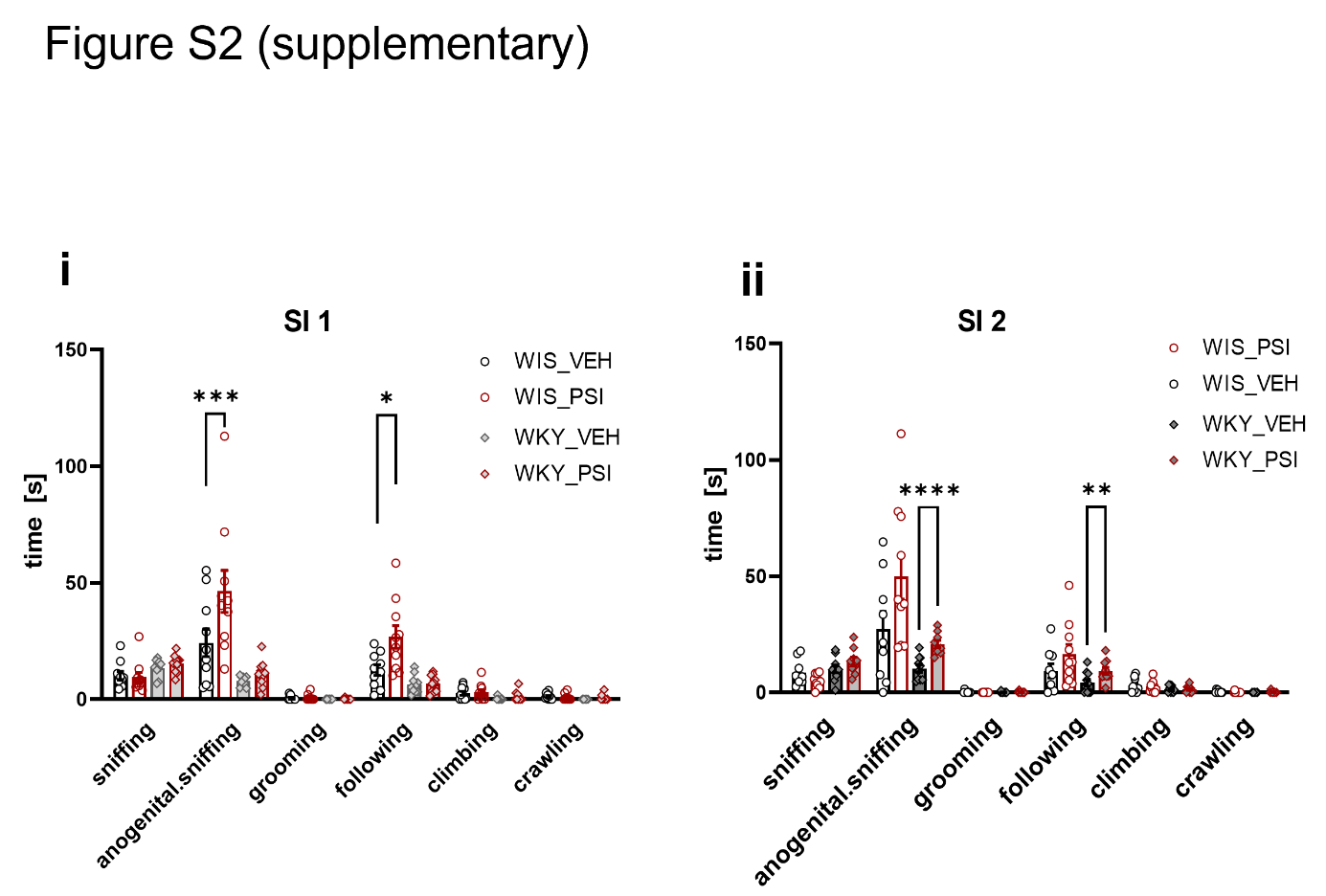


**Figure S2.** Time spent on specific behaviors during SI1 and SI2 tests. SI1 was performed 2 days after PSI administration (A), and SI2 was performed 8 days after PSI administration (B). WKY rats are depicted as rhomboid symbols and grey shaded bars, while WIS rats are represented as squared symbols and white bars. In SI1, there were significant increases in anogenital sniffing and following behavior after PSI administration in WIS rats (post hoc pairwise t-test comparisons*p < 0.05, ***p < 0.001). In SI2, there were significant increases in similar types of behavior in WKY rats (post hoc pairwise t-test comparisons**p < 0.01, ****p < 0.0001).


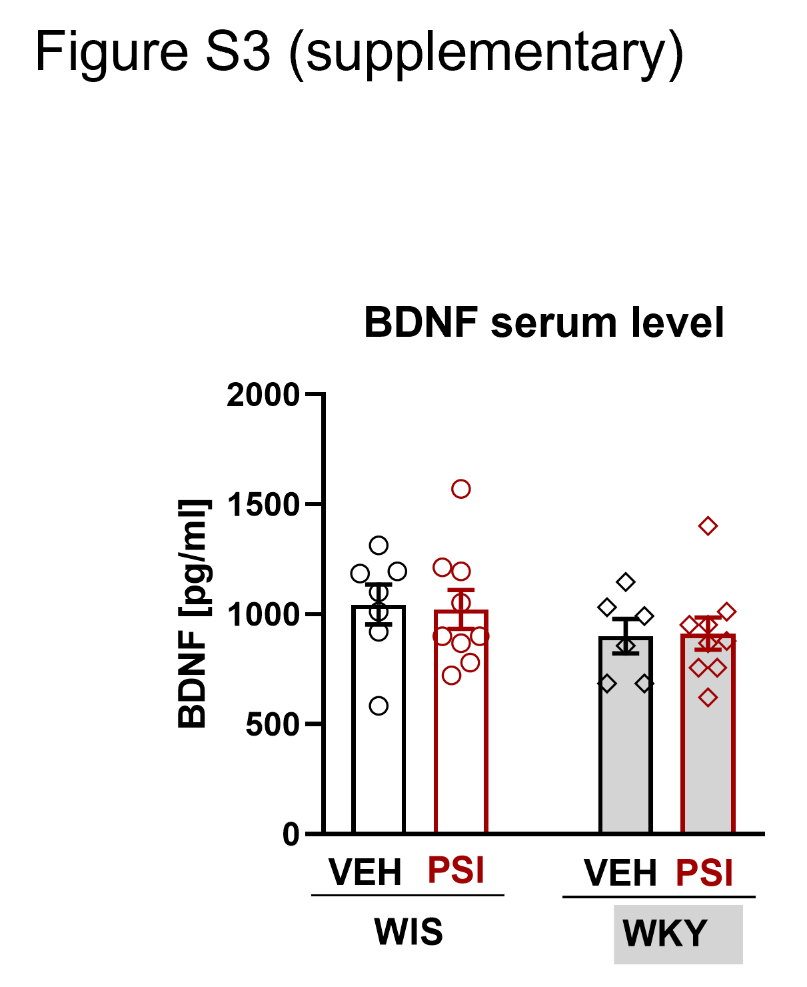


**Figure S3.** Total BDNF levels in the serum of WIS and WKY rats after PSI administration. BDNF serum levels were assessed 24 days after PSI administration in rats following behavioral procedures, with n = 7-10 rats per group. WKY rats are depicted as rhomboid symbols and grey shaded bars, while WIS rats are represented as squared symbols and white bars. No significant differences were found in serum levels either between WIS and WKY or after PSI treatment.


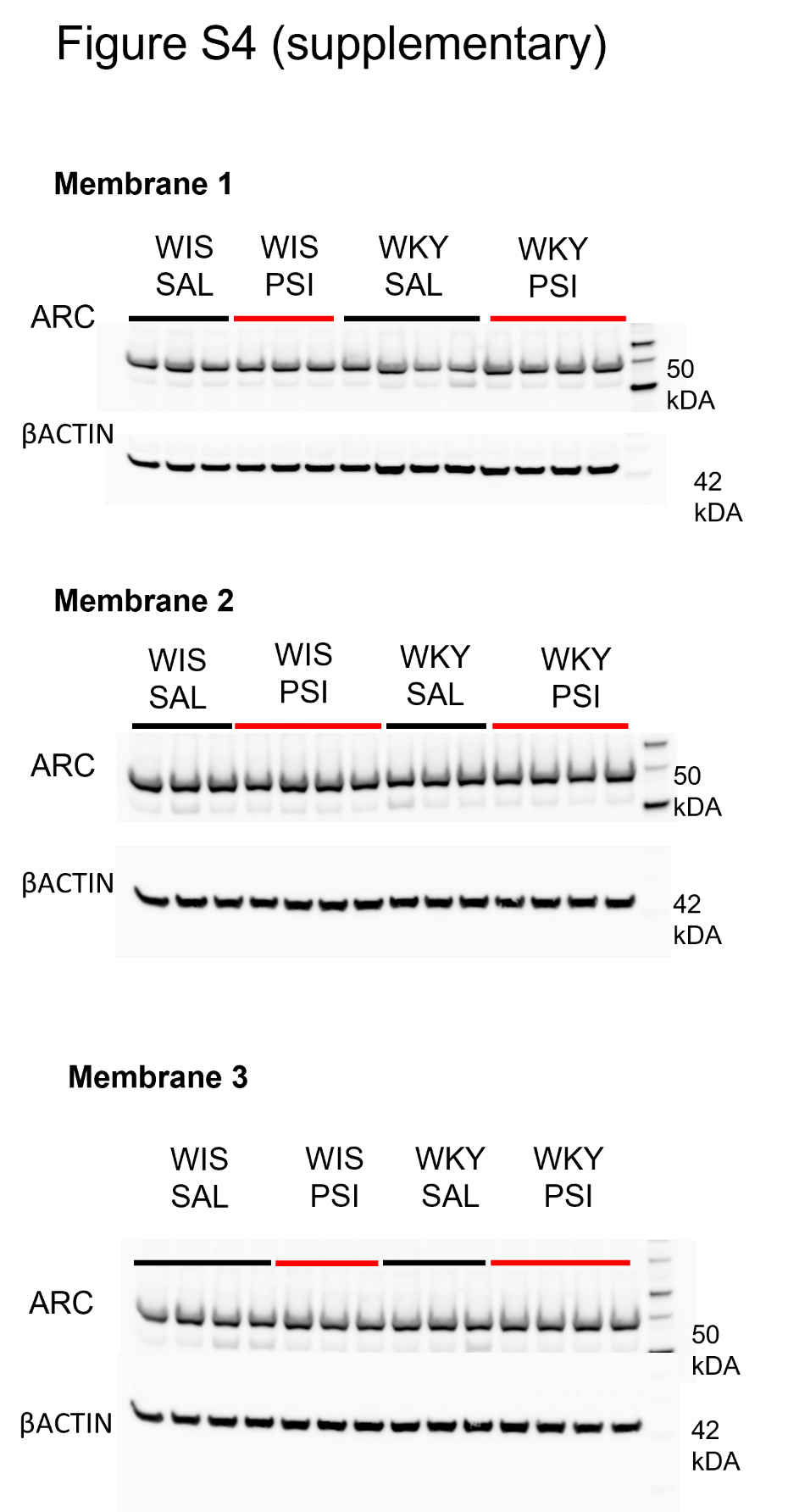


**Figure S4.** Western blot images for all individuals along with marked sample layout.

**Figure S5.** The photographs of paired objects used in both NORTs. The objects used in NORT 1 included a glass bulb filled with gravel and a plastic bottle filled with sand. In the second test (NORT 2), the set of objects was replaced with a new one (metal can and glass cylinder). The height of the objects was comparable (~12 cm), and they were of sufficient weight to prevent displacement by the animals.
